# AI-guided identification of natural CTSL inhibitors with therapeutic potential for renal injury

**DOI:** 10.1371/journal.pcbi.1014464

**Published:** 2026-07-10

**Authors:** Feier Ma, Qi Li, Sirui Zhou, Xiaoya Li, Jin-Kui Yang

**Affiliations:** 1 Beijing Key Laboratory of Diabetes Research and Care, Beijing Diabetes Institute, Beijing Tongren Hospital, Capital Medical University, Beijing, China; 2 Laboratory for Clinical Medicine, Capital Medical University, Beijing, China; University of Maryland School of Pharmacy, UNITED STATES OF AMERICA

## Abstract

Cathepsin L (CTSL) is a prominent therapeutic target for kidney injury, yet clinically available CTSL inhibitors remain limited. Here, we developed an artificial intelligence (AI)-assisted discovery strategy to identify novel CTSL inhibitors from a natural products library. Through a robust deep learning model and molecular docking, we screened 200 molecules from natural products library for experimental validation. Active candidates were further analyzed by molecular dynamics simulations to characterize binding modes and key CTSL-ligand interaction networks, followed by evaluation of therapeutic efficacy in kidney injury-relevant models. At a concentration of 100 µM, we found that 43 of them exhibited more than 50% inhibition of CTSL. Notably, nine molecules displayed over 90% inhibition and exhibited concentration-dependent effects. Molecular dynamics simulations indicated that Kuwanon G (KG), Iberverin, and Wighteone stably bind within the CTSL active site. In human renal cells, KG attenuated high glucose and high lipid induced inflammatory and injury responses. Collectively, these findings identify new CTSL inhibitors with therapeutic potential for renal injury and underscore the utility of AI-assisted strategies in accelerating drug discovery.

## Author summary

Computational approaches are increasingly used to speed up early-stage drug discovery, but many disease-relevant targets still lack effective inhibitors. Cathepsin L (CTSL) is an enzyme implicated in kidney injury and blocking CTSL has shown kidney-protective effects in studies. In this work, we combined artificial intelligence (AI) with laboratory experiments to search a natural products library for new CTSL inhibitors. AI models and molecular docking helped us narrow thousands of compounds to 200 candidates for testing. We then validated CTSL inhibition experimentally and used molecular dynamics simulations to understand how the most active compounds bind to CTSL. In human kidney cells exposed to high glucose and high lipid stress, the top candidate reduced injury-associated responses. Overall, our findings provide new CTSL inhibitor leads for kidney injury and demonstrate how AI can streamline the identification and characterization of bioactive molecules.

## 1. Introduction

Diabetic kidney disease (DKD) represents the primary contributor to end-stage renal disease (ESRD) worldwide and develops in approximately 40% of individuals with diabetes [1]. Although standard therapies including renin angiotensin aldosterone system (RAAS) blockade [2], sodium-glucose cotransporter-2 (SGLT2) inhibitors and glucagon-like peptide-1 (GLP-1) receptor agonists [3] and non-steroidal mineralocorticoid receptor antagonists (MRAs) like finerenone have demonstrated significant renal protective benefits and established a mechanistic rationale for pharmacological intervention, a considerable residual risk of progression persists [4], highlighting the pressing demand for novel therapeutic approaches.

Among the emerging targets, Cathepsin L (CTSL), a lysosomal cysteine protease, has garnered increasing attention for its multifaceted role in renal pathology. Beyond its canonical function in intracellular protein turnover, CTSL is implicated in critical processes underpinning kidney injury progression, including podocyte injury via cleavage of key structural proteins like dynamin [5]. In addition, CTSL global knockout mice showed an improved phenotype in the STZ-induced diabetic kidney disease model [6]. Crucially, evidence from both animal models and human studies indicates that CTSL expression and activity are markedly upregulated under diabetic conditions [6,7]. Our previous work further illuminated that hyperglycemia promotes the maturation and lysosomal translocation of CTSL, suggesting a direct link between diabetic stress and enhanced CTSL mediated proteolysis, which may exacerbate diabetic complications [8]. Despite these compelling insights, the translational potential of CTSL inhibition as a therapeutic strategy for kidney injury remains largely unexplored, presenting a significant knowledge gap and an opportunity for pharmacological intervention.

The prolonged use of conventional drugs often causes adverse effects and renal burden, highlighting the need for safer and more effective therapies. Due to their chemical heterogeneity and generally well-tolerated safety characteristics, natural compounds have re-emerged as promising sources for therapeutic development [9]. Various compounds—including flavonoids, phenolic acids, alkaloids, ginsenosides, isothiocyanates and polyphenols [10] have shown renoprotective effects in kidney injury models via anti-inflammatory, antioxidant, and anti-fibrotic actions. Although some of these agents inhibit CTSL, evidence for their kidney injury-specific efficacy remains limited. Thus, we applied a target-directed selection to identify natural compounds with stronger CTSL inhibition, clear binding characteristics, and favorable cytotoxicity and kidney injury-related profiles, leading to the identification of promising candidates such as KG.

The advent of artificial intelligence (AI) has revolutionized drug discovery by enabling the rapid and efficient screening of vast chemical libraries. Deep learning (DL) algorithms, in particular, can predict ligand-target interactions with remarkable accuracy, significantly accelerating the initial hit identification phase [11]. The combination of AI with structural computation methods—including ligand-receptor conformational mapping and time-resolved simulation of atomic interactions—enables a systematic and mechanism-oriented paradigm for lead compound optimization. Chemprop [12] is a deep learning-based tool designed for predicting molecular properties and informing drug discovery.

Our previous research [13,14] constructed a structure-activity prediction framework based on the graph neural network architecture implemented in Chemprop, enabling quantitative estimation of compound-level inhibitory activity against members of the Cathepsins. This model was subsequently deployed to interrogate natural product libraries, facilitating in silico prioritization of candidate inhibitors. Collectively, these efforts underscore the translational value of data-driven AI methodologies in accelerating target-oriented drug discovery pipelines. This integrated computational approach enables efficient prioritization of promising candidates for experimental validation, thereby optimizing resource use and enhancing the probability of successful discovery. Guided by this rationale, we hypothesized that an AI-based screening approach could efficiently identify novel CTSL-targeting natural products for kidney injury. Here, we applied AI-based CTSL inhibitor prediction followed by MD simulation, and targeted in vitro assays to validate prioritized candidates. This strategy successfully identified a natural CTSL inhibitor that markedly attenuated high glucose and high lipid-induced renal cellular injury.

Taken together, our findings underscore that AI-assisted screening can markedly accelerate early-stage drug discovery by efficiently prioritizing bioactive candidates for experimental validation, while yielding promising CTSL-targeted therapeutic leads for kidney injury. Moreover, this integrated computational-experimental workflow provides a scalable framework for drug discovery and mechanistic characterization.

## 2. Materials and methods

### 2.1. Deep learning model training and predictions

We used a deep learning model to identify putative cathepsin L (CTSL) inhibitors from natural products. The model used a Message Passing Neural Network (MPNN) architecture, as implemented in the Chemprop software package [12], which predicts the probability of a molecule inhibiting CTSL activity. The training dataset was obtained from the PubChem database using CTSL-associated records (Gene ID: 1514), comprising 2062 compounds annotated as active CTSL inhibitors and 58,005 compounds annotated as inactive (S1 Table). Using RDKit [15], each molecule was converted from its SMILES representation into a molecular graph, as described in previous studies [16]. The dataset was randomly divided into training, validation, and test sets at a ratio of 80:10:10. The model was trained for 30 epochs using the training set, and its performance was evaluated on the validation set at the end of each epoch. After training, the model checkpoint with the best validation performance was selected and further evaluated on the independent test set. The trained model generated a probability score ranging from 0 to 1 for each input molecule, with higher values indicating a greater predicted likelihood of CTSL inhibition.

To improve model performance, we applied a Bayesian hyperparameter optimization strategy. Bayesian optimization is a principled approach that uses a probabilistic surrogate model of the objective function to guide the exploration of the hyperparameter space based on prior observations [17]. This strategy enables more efficient identification of optimal hyperparameter configurations than exhaustive or random search. In this study, 20 iterations of Bayesian optimization were performed to optimize key model parameters, including the number of message-passing steps, the number of feedforward classifier layers, the hidden dimensionality, and the dropout probability.

The prediction dataset consisted of 4,946 natural products from the Med Chem Express HY-L021 library. To ensure complete separation between the training and prediction datasets, a deduplication procedure was performed. Canonical SMILES were generated using the RDKit canonicalization algorithm and used as structure identifiers. Compounds in the prediction dataset with canonical SMILES identical to those in the training dataset were removed, resulting in 4,464 unique compounds for model prediction. The optimized model then assigned each compound an inhibition probability score on a scale from 0 to 1, with higher scores indicating a greater predicted likelihood of CTSL inhibitory activity.

### 2.2. Receptor protein preparation

The receptor structure of CTSL was prepared using the *prepare_receptor4.py* module in Auto Dock Tools (ADT) [18]. The three-dimensional (3D) coordinates of CTSL were obtained from the Protein Data Bank (PDB entry: 5MQY) [19]. Prior to docking, the receptor structure underwent standard preprocessing procedures, including explicit addition of hydrogen atoms, consolidation of nonpolar hydrogens, and assignment of Gasteiger partial charges together with ADT atom type parameters to ensure compatibility with subsequent molecular docking simulations. The structure was carefully inspected for completeness and corrected for any missing atoms. The finalized receptor model was then converted to the.*pdbqt* format for subsequent molecular docking analyses. The resulting protein-ligand complexes were further refined by restrained geometry minimization using the OPLS3 force field [20].

### 2.3. Ligands preparation

We employed the gen3D (generate 3D coordinates) module of Open Babel (version 2.3.0) for the conversion of chemical structures in the Med Chem Express HY-L021 library from SMILES format to mol2 format, rendering them suitable for subsequent docking analyses [21]. Ligand preparation was performed using the *prepare_ligand4.py* utility in ADT. During preprocessing, rotatable bonds were identified, Gasteiger partial charges were computed, and atomic parameters were assigned. Each ligand was subsequently optimized and converted to the.*pdbqt* format for molecular docking analysis.

### 2.4. Molecular docking

Molecular docking between CTSL and the candidate ligands was performed using AutoDock Vina. The exhaustiveness value was set to 8 to ensure adequate sampling of conformational space. The docking grid was centered at coordinates (−9.25, 5.75, 10.6), with dimensions of 20 Å × 20 Å × 20 Å, encompassing the catalytic pocket of CTSL. The docking workflow was designed to explore the optimal ligand-protein interactions and determine the energetically most favorable binding conformations. The pose exhibiting the lowest binding free energy was selected for subsequent analysis. Visualization and examination of the binding orientations and key residue interactions were conducted using PyMOL, providing structural insights into the CTSL-ligand complex. In addition, correlation analyses were performed to evaluate the relationships among predicted activity scores, docking rank, and experimentally measured CTSL activity.

### 2.5. CTSL activity test

The inhibitory potency of candidate compounds against CTSL was evaluated using a fluorometric enzyme activity assay. Reactions were carried out in a 100 μL system containing MES buffer (50 mM, pH 5.5) supplemented with 2.5 mM DTT and 0.5 mM EDTA. Each reaction mixture consisted of 1 μL of CTSL enzyme (5 mg/L; Abcam, ab157067), 2 μL of the fluorogenic substrate Z-FR-AFC (0.5 mM; MCE, HY-P4217), 1 μL of compound solution (serially diluted in DMSO), and 96 μL of assay buffer. Time-resolved fluorescence signals were recorded using a Spark multimode microplate reader (Tecan), with the instrument temperature controlled at 37 °C. Fluorescence detection was conducted at an excitation wavelength of 380 nm and an emission wavelength of 460 nm. Fluorescence intensity was recorded at 1-minute intervals over a 60-minute period. The initial reaction velocity was calculated from the linear phase of fluorescence increase and used to quantify enzymatic activity. For inhibitory concentrations (IC_50_) determination, compounds were tested at concentrations ranging from 100 μM to 0.01 μM using a semi-logarithmic dilution series. E64d was included in parallel as a positive control for assay performance. Percentage inhibition was calculated relative to vehicle-treated controls. Concentration-inhibition relationships were modeled by applying nonlinear regression to a four-parameter logistic equation, enabling calculation of IC_50_.

### 2.6. Molecular dynamics simulation studies

To evaluate the stability and binding characteristics of the CTSL-lead compound complex [22], molecular dynamics (MD) simulations were performed using GROMACS 2021.3 [23]. The initial complex structure was derived from the docking pose with the most favorable docking score [24]. The AMBER99SB-ILDN all-atom force field was applied to the protein [25] and the ligand topology and force-field parameters were generated using the Sobtop program with the General AMBER Force Field (GAFF) [26,27]. The complex was solvated using the TIP3P water model under periodic boundary conditions [28].

Energy minimization was first performed using the steepest descent algorithm for a maximum of 50,000 steps, or until the maximum force was below 1000 kJ mol ⁻ ¹ nm ⁻ ¹. The minimized system was then equilibrated in two stages. First, a 100-ps NVT equilibration was performed at 310 K using the velocity-rescaling thermostat [29]. Second, a 100 ps NPT equilibration was performed at 310 K and 1 bar using the velocity-rescaling thermostat and the Parrinello–Rahman barostat [30]. Position restraints were applied during the equilibration stages as defined in the topology. The production MD simulation was subsequently carried out for 100 ns with a 2-fs time step using the leap-frog integrator. Covalent bonds involving hydrogen atoms were constrained using the LINCS algorithm [31]. Long-range electrostatic interactions were treated using the particle mesh Ewald method [32].

The stability of the complex was assessed by calculating the root-mean-square deviation (RMSD) of the protein backbone and ligand heavy atoms relative to the initial conformation. Protein-ligand interactions were further analyzed using the *gmx_MMPBSA* tool [33] to estimate the binding free energy (ΔG_bind_) based on the molecular mechanics/Poisson-Boltzmann surface area (MM/PBSA) approach. The energy components-including van der Waals (ΔE_vdw_), electrostatic (ΔE_ele_), and solvation (ΔG_sol_) contributions were decomposed to identify key residues contributing to binding.

### 2.7. Cell culture and reagents

Human renal tubular (HK-2) cells were cultured in low-glucose Dulbecco’s Modified Eagle Medium (DMEM), supplemented with 10% fetal bovine serum (FBS) and 1% penicillin-streptomycin (Pen-Strep) to prevent bacterial contamination. Cell cultures were maintained under standard conditions in a humidified incubator set at 37 °C with 5% CO_2_. Culture medium was refreshed at 48-hour intervals to sustain optimal growth. Upon reaching approximately 70–80% confluence, cells were subjected to routine passaging. Briefly, adherent cells were enzymatically dissociated with 0.05% trypsin-EDTA and subsequently replated at a defined seeding density for continued expansion. Iberverin (Iber, HY-121204), Mulberrin (HY-N3513), (-)-Alkannin (HY-N6012), 8-Methylsulfinyloctyl isothiocyanate (HY-115770), Kuwanon G (KG, HY-N4247), Gamma-Mangostin (HY-N1957), Wighteone (Wig, HY-N1073), Glycyrrhisoflavone (HY-N3962), Glabrol (HY-N4193), E64d (HY-100229) and other compounds were purchased from Med Chem Express (MCE) (Monmouth Junction, NJ, USA).

### 2.8. Cell viability assay

Cell viability was assessed by the CCK-8 assay (Beyotime, China) to evaluate the effects of Iber, KG and Wig on HK-2 cells. HK-2 cells were plated into 96-well plates at a density of 5 × 10^4^ cells per well and cultured overnight to permit attachment. Cells were then exposed to graded concentrations of the test compound for 24 h. Following treatment, 10 μL of CCK-8 reagent was introduced into each well, and incubation was continued for 2 h at 37 °C to allow color development. Optical density was recorded at 450 nm using a microplate reader. Viability was calculated as the percentage of absorbance relative to untreated controls, which were normalized to 100%. All assays were performed with three independent biological replicates, each containing technical triplicates.

### 2.9. Determination of injury-related protein expression in HK-2 cells

Upon attaining roughly 80% confluence, the cultured cells were allocated into the experimental groups as outlined below for subsequent interventions. The experimental groups included the low-glucose (Control) group (5 mM glucose), the high glucose and high lipid (HGL) group (35 mM glucose and 400 µM PA), the drug control group (DMSO and HGL), and the drug treatment group (Compound and HGL). Following 48 h exposure to the indicated pharmacological treatments or HGL stimulation, cells were harvested for total protein isolation.

### 2.10. Western blotting

Total protein was extracted from HK-2 cells using RIPA lysis buffer supplemented with phenylmethylsulfonyl fluoride (PMSF) and a protease inhibitor cocktail (PI). Following centrifugation, clarified lysates were harvested and total protein concentrations were quantified using a bicinchoninic acid (BCA) assay. Equivalent amounts of protein (30 μg per lane) were combined with SDS loading buffer, heat-denatured at 100 °C for 5 min, and resolved by electrophoresis on 7.5-12% precast SDS-polyacrylamide gels. Proteins were subsequently transferred onto methanol-activated PVDF membranes for immunoblot analysis.

After blocking to prevent nonspecific binding, membranes were incubated with the indicated primary antibodies at 4 °C overnight, followed by exposure to species-appropriate secondary antibodies. Immunoreactive bands were detected using an enhanced chemiluminescence system and visualized with the LAS-500 platform (Fujifilm). Densitometric quantification was performed using ImageJ.

Primary antibodies included Tubulin (1:1000), HSP90 (1:1000), TGF-β1 (1:1000), NGAL (1:2000), p-AKT (1:1000), and CTSL (1:1000).

### 2.11. Quantitative real-time polymerase chain reaction (RT-qPCR)

Total RNA was isolated from cultured cells using a commercial RNA extraction kit in accordance with the manufacturer’s protocol. Complementary DNA (cDNA) synthesis was carried out with a reverse transcription premix (Accurate Biology, AG11706, China). Gene-specific primers were designed and synthesized by Sangon Biotech, as listed in Table 1.

**Table 1:** Sequence of quantitative real-time PCR forward primers and reverse primers.

Gene	Primer(F)	Primer (R)
β-actin	5’-GCACTCTTCCAGCCTTCCTT-3’	5’-AGGTCTTTGCGGATGTCCA-3’
CTSL	5’-AAACTGGGAGGCTTATCTCACT-3’	5’-GCATAATCCATTAGGCCACCAT-3’
NGAL	5’-CCCTTGTCTCTAAGGATTGGCG-3’	5’-CCAGCCATAAAACAGGAAGAGGA-3’
TNF-α	5’-CGAGTGCAAGCCTGTAGC-3’	5’-GGTGTGGGTGAGGAGCACAT-3’
AKT	5’-AAGAAGGAGGTCATCGTCGC-3’	5’-CTTGAGGGCCGTAAGGAAGG-3’

RT-qPCR was conducted using a SYBR Green master mix (Yeasen Biotech, 11201ES08, China) under the following thermocycling parameters: initial denaturation at 95 °C for 30 s, followed by 40 amplification cycles of 95 °C for 10 s, 60 °C for 30 s, and 72 °C for 30 s. Relative mRNA expression levels were calculated using the 2^−ΔΔCT^ method, and statistical analyses were performed with GraphPad Prism 8.0.2.

### 2.12. Enzyme kinetics

To elucidate the inhibition modality, initial reaction rates were quantified across a gradient of substrate concentrations 50 µM, 25 μM, 12.5 μM, 6.25 μM, 3.125 μM, 1.5625 μM Z-FR-AFC in the presence of different inhibitor concentrations 100 μM, 30 μM, 10 μM, 3 μM, 1 μM, 0 μM. Data were fitted to the Michaelis-Menten equation to derive Michaelis constant (K_m_) and maximal catalytic rate (V_max_) values, and the inhibition type (competitive, non-competitive, or uncompetitive) was identified using Lineweaver-Burk plots.

### 2.13. LC–MS analysis of intracellular compounds

Kuwanon G (KG), Iberverin, and Wighteone were obtained from Med Chem Express with purity ≥98%. Berberine (BBR) was used as an internal standard (IS) and purchased from Bide Pharm. LC-MS grade methanol and formic acid were purchased from Fisher. Ultrapure water was generated using a Milli-Q system. Human renal HK-2 cells were treated with KG, Iberverin, or Wighteone at 10 μM for 48 h under identical conditions. After treatment, cells were rapidly washed three times with ice-cold PBS to remove extracellular compounds. Cells were lysed using methanol/water (80:20, v/v) mixture to precipitate proteins and extract intracellular metabolites. LC-MS analysis was performed on a Thermo Orbitrap Exploris 480 mass spectrometer with a Waters C18 column. Quantification was performed using calibration curves constructed by plotting the peak area ratio of analyte to internal standard (BBR) versus nominal concentration.

### 2.14. Statistical analysis

Kinetic parameters were derived by fitting the experimental velocity data to the Michaelis-Menten model using nonlinear regression, allowing estimation of the K_m_ and V_max_. Concentration-response relationships were analyzed through nonlinear regression of log-transformed inhibitor concentrations against normalized activity values, from which IC_50_ values corresponding to 50% inhibition were obtained. Graphical presentation and statistical computations were conducted with GraphPad Prism 8.0.2 (GraphPad Software, San Diego, CA, USA). Quantitative data are reported as mean ± standard error of the mean (SEM). Intergroup differences involving two independent cohorts were evaluated using a two-sided, unpaired Student’s *t*-test. For experiments comprising more than two conditions, statistical inference was performed by one-way analysis of variance (ANOVA), followed by Tukey’s multiple-comparison procedure to control for type I error. A threshold of *p* < 0.05 was predefined as indicative of statistical significance. Correlation analysis between predicted activity, docking rank, and experimentally measured CTSL activity was conducted using Spearman’s rank correlation coefficient in SPSS (version 27.0; IBM Corp., Armonk, NY, USA).

## 3. Results

**Fig 1:**
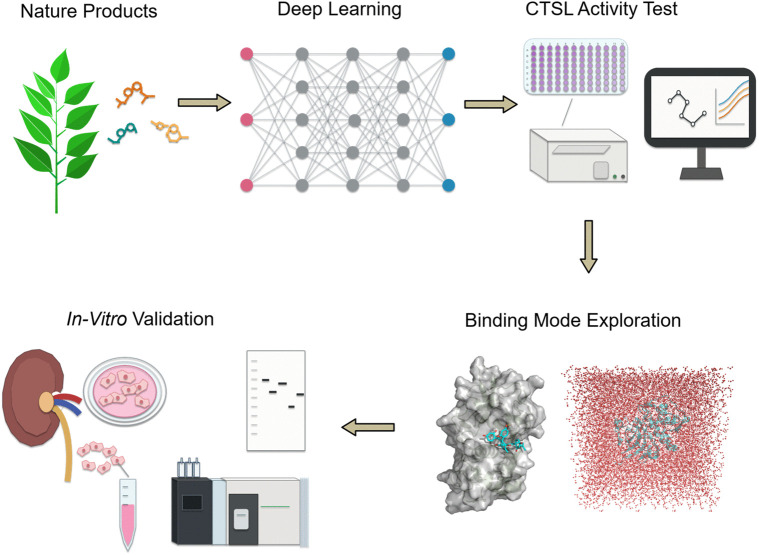
Overall workflow. A curated natural products library containing 4,946 structurally unique compounds was analyzed using an AI-driven screening strategy designed to rapidly prioritize molecules with potential CTSL inhibitory activity from a chemically diverse compound space. The AI-prioritized candidates were further refined by molecular docking, and 200 compounds were selected for CTSL enzymatic activity testing. Active compounds were then subjected to binding-mode exploration, including protein–ligand interaction analysis and molecular dynamics simulations, to evaluate the stability of CTSL–ligand complexes and infer key interaction mechanisms. Candidate inhibitors with favorable enzymatic activity and computational binding profiles were subsequently evaluated in in-vitro kidney injury models. Finally, the lead compound with the strongest protective effect was subjected to enzyme kinetic analyses to determine its inhibitory mode and kinetic parameters.

The overall workflow of this study, integrating AI-driven screening, computational validation, and experimental evaluation to identify natural CTSL inhibitors with therapeutic potential for renal injury, is illustrated in Fig 1.

### 3.1. CTSL inhibitor model training and prediction

Message Passing Neural Networks (MPNNs) constitute a family of deep neural architectures tailored for graph-structured inputs. Within the context of cheminformatics, small molecules are encoded as topological graphs in which atoms serve as vertices and covalent bonds define the connecting edges. This architecture enables MPNNs to capture complex topological and electronic features that underlie molecular bioactivity, thereby facilitating accurate prediction of ligand-target interactions. In this study, we applied an MPNN model previously developed and validated by our research team to identify potential CTSL inhibitors [34,35]. The model was trained on a curated PubChem BioAssay dataset containing approximately 60,000 compounds annotated for CTSL inhibitory activity. After optimization, the model achieved robust predictive performance with a ROC-AUC of 0.93, indicating high classification accuracy between active and inactive molecules.

**Fig 2:**
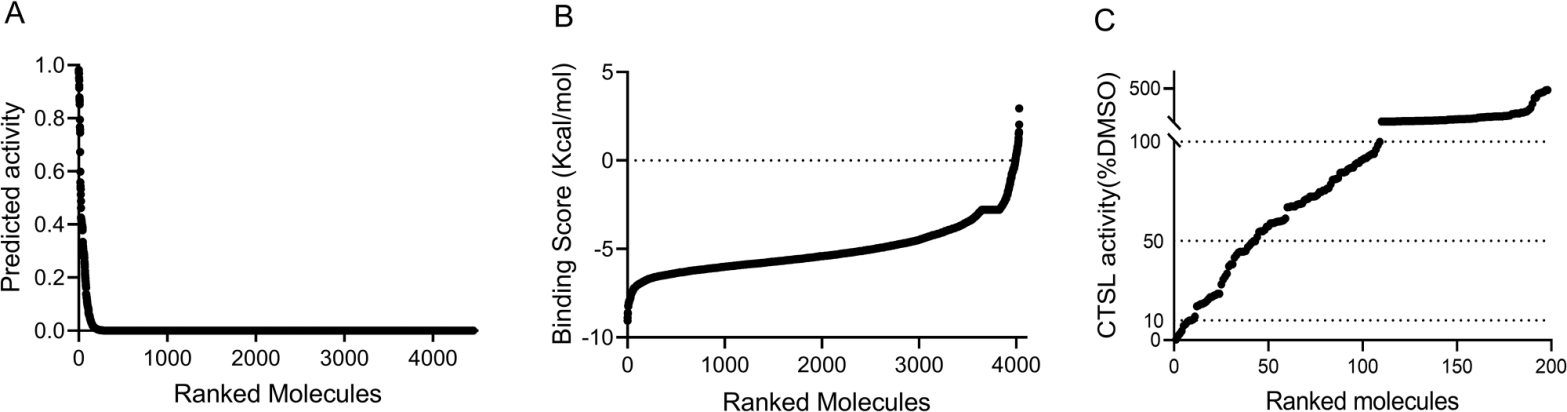
CTSL inhibitors prediction and enzyme activity test. **A,** Rank-ordered CTSL inhibitor prediction scores for 4,464 compounds, where higher prediction scores indicate a greater probability of inhibitory activity against CTSL. **B,** The molecular docking scores of 4031 natural products to CTSL (PDB 5MQY). **C,** The top 200 molecules were selected to verify their inhibitory effects against CTSL in a cell-free system at a concentration of 100 μM.

We subsequently employed this optimized model to screen the natural products library—Med Chem Express HY-L021—comprising 4,946 natural compounds with diverse chemical scaffolds. To ensure data integrity, compounds with identical molecular graphs to those in the training dataset were excluded, yielding 4,464 structurally unique molecules. Then, we determined the prediction scores for each molecule, and each molecule was ranked based on its probability score representing its predicted inhibitory potential toward CTSL (Fig 2A).

### 3.2. Molecule docking

**Fig 3:**
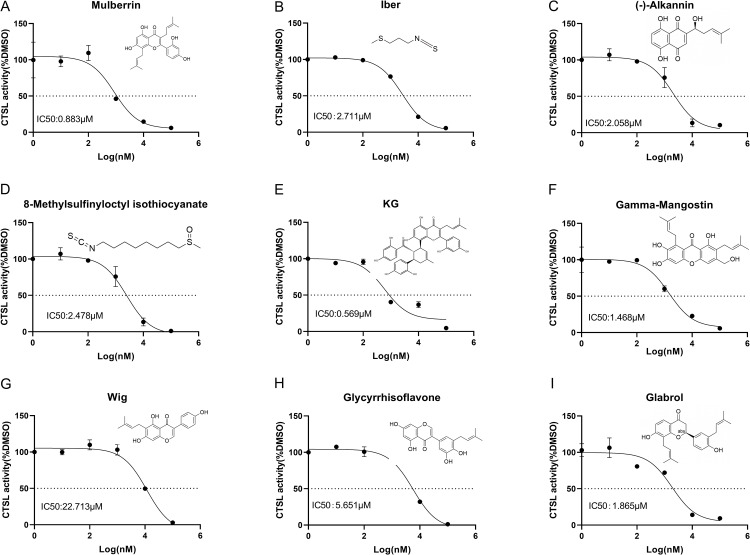
Half-maximal inhibitory concentration (IC_50_) determination. **A-I,** Dose-response curves of nine natural CTSL inhibitors. Mulberrin **(A)**, Iber **(B)**, (-)-Alkannin **(C)**, 8-Methylsulfinyloctyl isothiocyanate **(D)**, KG **(E)**, Gamma-Mangostin **(F)**, Wig **(G)**, Glycyrrhisoflavone **(H)**, and Glabrol **(I)**, which exhibited >90% inhibition of CTSL activity at 100 μM in the primary screen, were further evaluated to determine their half-maximal inhibitory concentrations (IC_50_) in an in vitro enzymatic assay. Dose-response curves were generated by nonlinear regression fitting of the data (black lines). Data are presented as mean ± SEM from a representative experiment performed in triplicate.

Molecular docking enables the prediction of interactions between small molecules and their respective targets, typically proteins, facilitating an evaluation of the molecule’s binding affinity and conformation. Following the predictions from the MPNNs model, we conducted molecular docking using AutoDock Vina, employing human CTSL crystal structures obtained from the PDB database (PDB code: 5MQY). The 3D molecular structures were generated using the gen3D module of Open Babel (version 2.3.0) [21]. The docking results were ranked according to the docking score with CTSL (Fig 2B). As 3D structure generation was unsuccessful for a subset of compounds, a total of 4,031 compounds with valid 3D conformations were retained for downstream analysis. We aggregated the predictive scoring ranks of these molecules with their respective docking scores to establish a composite ranking. From this integrated ranking, the top 200 molecules were selected for subsequent experimental validation. We administered a uniform dose of 100 μM to evaluate their potential in inhibiting CTSL activity. The assay results showed that 43 out of the 200 molecules exhibited more than 50% inhibition of CTSL. Notably, the most effective inhibitors, including Mulberrin, Iberverin (Iber), (-)-Alkannin, 8-Methylsulfinyloctyl isothiocyanate, Kuwanon G (KG), Gamma-Mangostin, Wighteone (Wig), Glycyrrhisoflavone, and Glabrol demonstrated inhibition efficiencies exceeding 90% (Fig 2C, S2 Table). To provide a positive control for assay performance, we first tested E64d, a well-characterized cysteine protease inhibitor with CTSL inhibitory activity, under the same fluorometric assay conditions. E64d inhibited recombinant CTSL activity in a concentration-dependent manner, with an IC_50_ value of 7.738 μM (S1A-S1B Fig). These results provided a reference benchmark for CTSL inhibition and supported the responsiveness of the enzymatic assay system. We then tested a range of concentrations of the top 9 most effective inhibitors for further confirmation and determination of the IC_50_. Notably, all 9 molecules inhibited CTSL activity in a concentration-dependent manner (Figs 3A–3I, S2). KG, Iber, and Wig exhibited the lowest IC_50_ values among the tested compounds. Therefore, they were selected as lead candidates for subsequent mechanistic and functional studies.

To assess whether the in silico prioritization metrics were associated with the experimental screening results, we performed correlation analyses between AI-predicted activity scores, docking rank, and experimentally measured residual CTSL activity. A statistically significant negative correlation was observed between AI-predicted activity score and residual CTSL activity (rs = -0.177, *p* = 0.012). The magnitude of this correlation was modest, indicating that AI-predicted activity scores explained only a limited proportion of the variability observed in the enzymatic assay. Nevertheless, the negative trend suggests that compounds assigned higher activity scores were, on average, more likely to exhibit stronger CTSL inhibition.

In contrast, docking rank showed no significant correlation with residual CTSL activity (rs = 0.022, *p* = 0.753). While docking rank alone was therefore not predictive of inhibitory potency in this dataset, docking remained a valuable complementary tool by incorporating structural information, eliminating compounds with unfavorable binding orientations, and providing mechanistic insight into ligand–CTSL interactions through binding-mode and molecular dynamics analyses. Overall, these results indicate that the AI model provided modest but statistically supported predictive information beyond docking-based ranking alone.

**Fig 4:**
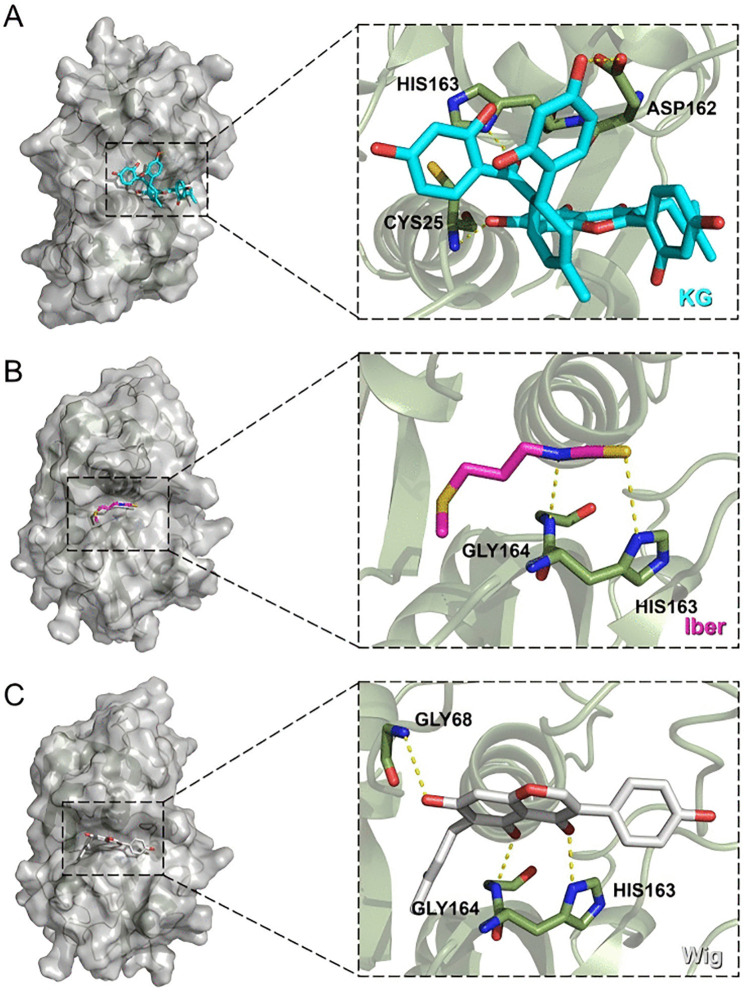
The docking result of KG, Iber and Wig in the CTSL structural crystal. **A,** Docking interactions of KG in the active sites of CTSL. KG is represented as sticks with carbon atoms in cyan. CTSL is shown as a green cartoon with a light grey surface. **B,** Docking interactions of Iber in the active sites of CTSL. Iber is represented as sticks with carbon atoms in magenta. CTSL is shown as a green cartoon with a light grey surface. **C,** Docking interactions of Wig in the active sites of CTSL. Wig is represented as sticks with carbon atoms in grey. CTSL is shown as a green cartoon with a light grey surface. Short intermolecular contacts with distances of < 3.5 Å between ligands and receptor are depicted as yellow dashed lines.

To analyze the binding states of KG, Iber, and Wig with the CTSL active site, we generated a three-dimensional (3D) representation illustrating their interactions. KG forms three hydrogen bonds with Cys25, Asp162, and His163 residues of CTSL (Fig 4A), Iber forms two hydrogen bonds with His163 and Gly164 residues of CTSL (Fig 4B), Wig forms three hydrogen bonds with Gly68, His163, and Gly164 residues of CTSL (Fig 4C).

### 3.3. Molecular dynamics simulation

Molecular docking provides only a static snapshot of protein-ligand interactions and cannot capture the dynamic behavior of the complex in a solvated environment. To address this limitation and gain insight into the conformational flexibility, binding stability, and persistence of interactions over time, we conducted a molecular dynamics (MD) simulation using the AMBER99SB-ILDN force field. MD simulations allow the evaluation of protein-ligand stability, identification of key interactions under physiological-like conditions, and assessment of conformational changes that may affect inhibitor efficacy [13].

**Fig 5:**
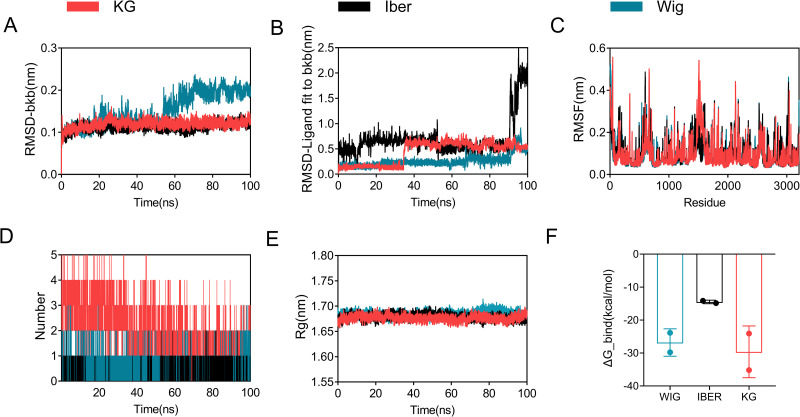
Molecular dynamics simulation results of KG, Iber and Wig. **A-E,** RMSD-bkb **(A)**, RMSD-Ligand **(B)**, RMSF **(C)**, H-bond (D) and radius of gyration of protein (E) for ligand-receptor complex. Red represents the KG-CTSL complex, black represents the Iber-CTSL complex, blue represents the Wig-CTSL complex. **F,** Block analysis of MM/PBSA binding free energies. The 100 ns trajectories were divided into two blocks, 0–50 ns and 50–100 ns. Each dot represents the mean ΔG_bind_ value from one block. Bars represent the average of the two block means, and error bars indicate the standard deviation of the two block means, equivalent to |mean0–50 ns − mean50–100 ns|/√2.

To evaluate the dynamic stability of the complexes under physiological conditions, 100 ns MD simulations were performed at 310 K and 1 bar (S3A-S3I Fig). Backbone RMSD analysis revealed that CTSL maintained overall structural stability throughout the 100 ns MD simulations. The mean RMSD-bkb values were 0.12 nm for the KG–CTSL complex, 0.11 nm for the Iber–CTSL complex, and 0.15 nm for the Wig–CTSL complex. The KG–CTSL and Iber–CTSL systems rapidly reached stable RMSD plateaus of approximately 0.11–0.12 nm, whereas the Wig–CTSL complex exhibited a moderate increase after 50 ns and subsequently stabilized at around 0.20 nm during the late stage of the simulation. Nevertheless, the backbone RMSD values of all systems remained below 0.24 nm, indicating that ligand binding did not induce substantial global conformational instability in CTSL (Fig 5A). After least-squares fitting of the receptor backbone, ligand RMSD analysis revealed distinct binding dynamics among the three complexes. For the KG–CTSL complex, although the ligand RMSD increased moderately after approximately 35 ns, it subsequently stabilized around 0.55–0.60 nm, indicating that KG achieved a stable binding pose after conformational adaptation within the active site. The Iber ligand displayed a more pronounced RMSD increase during the final 10 ns, reaching 1.8917 nm at 100 ns, suggesting substantial pose rearrangement or possible partial displacement from the binding pocket. In contrast, Wig exhibited the lowest average ligand RMSD of 0.2547 nm, indicating a highly stable binding orientation throughout most of the simulation. Together, these results suggest that Wig and KG maintained relatively favorable binding stability, whereas Iber showed reduced ligand pose stability during the late stage of the trajectory (Fig 5B). Root-mean-square fluctuation (RMSF) analysis, which reflects residue mobility and structural integrity, showed that all residues exhibited RMSF values below 0.55 nm in the presence of ligands (Fig 5C).

We then quantified hydrogen bonds between CTSL and the ligands over the trajectories using the Gromacs g_hbond tool. The results showed that up to three hydrogen bonds could form between each ligand and CTSL. Iber and Wig maintained 1–2 hydrogen bonds stably throughout the simulations, whereas KG consistently formed 2–4 hydrogen bonds over the simulation period (Fig 5D). In addition, the radius of gyration of each complex remained within a reasonable range, further supporting the global stability of the ligand-protein complexes (Fig 5E).

Binding free energy represents the overall energetic change upon ligand binding and reflects the strength and stability of the interaction. To assess the stability of the MM/PBSA estimates, ΔG_bind_ was calculated at 1 ns intervals throughout the 100 ns MD simulations (S3J Fig). The results indicated that all three CTSL–ligand complexes were consistent with energetically favorable ligand binding throughout the simulations. Nevertheless, the profiles revealed ligand-dependent temporal variations. KG displayed more favorable binding free energy during the first half of the trajectory but shifted toward less favorable values after approximately 40 ns, whereas Wig maintained relatively favorable ΔG_bind_ values over most of the simulation. Iber showed less favorable but comparatively stable ΔG_bind_ values.

To account for this temporal variation, we performed a two-block analysis by dividing the 100 ns trajectory into 0–50 ns and 50–100 ns segments. The mean ΔG_bind_ was calculated for each block, and the error bars were defined as the standard deviation of the two block means, equivalent to |mean_0–50 ns_ − mean_50–100 ns_|/√2. This block-based uncertainty was used instead of the standard deviation of all frame-wise MM/PBSA values. The averaged ΔG_bind_ values were approximately −29.8 ± 7.8 kcal/mol for KG, −14.6 ± 0.6 kcal/mol for Iber, and −27.1 ± 4.0 kcal/mol for Wig (Fig 5F). These results indicate that KG and Wig have more favorable predicted binding free energies than Iber, although the relatively large block-analysis uncertainties, particularly for KG, suggest that the relative difference between KG and Wig should be interpreted with caution.

### 3.4. KG attenuates HGL induced renal injury in HK-2 cells

Previous evidence implicates CTSL in both the initiation and progression of kidney injury and that CTSL inhibition confers marked renoprotective effects [6,36]. Accordingly, we hypothesized that KG, Iber, and Wig would also exert renoprotective effects. We first assessed the cytotoxicity of KG, Iber, Wig, and the reference inhibitor E64d in human renal HK-2 cells using the CCK-8 assay. After 24 h of incubation, KG, Iber, and Wig at 100 μM exhibited significant cytotoxicity, whereas KG and Iber at 30 μM and Wig at 10 μM did not affect cell viability. In parallel, E64d was evaluated to determine an appropriate concentration for use as a positive control in the cellular assay. E64d at 100 μM reduced HK-2 cell viability, whereas concentrations from 1 to 30 μM showed no obvious cytotoxic effect. Therefore, 30 μM KG, 30 μM Iber, 10 μM Wig, and 30 μM E64d were selected for subsequent experiments (S4 Fig).

**Fig 6:**
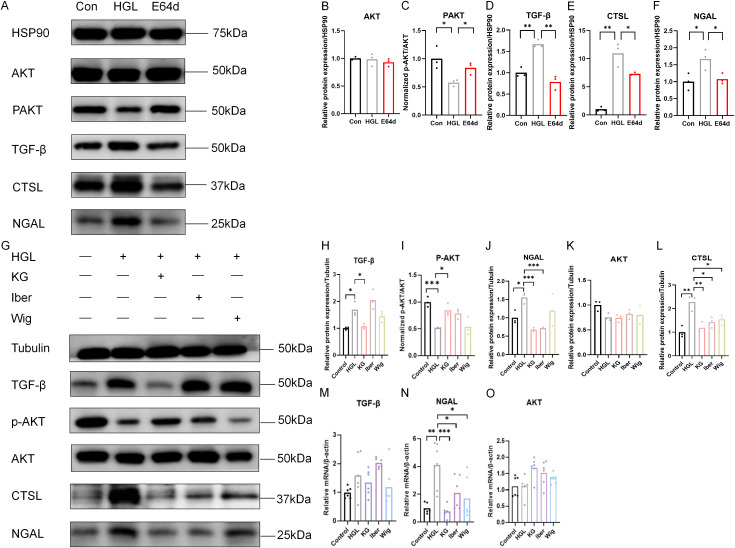
Validation of CTSL inhibition and renoprotective effects of E64d and AI-identified CTSL inhibitors in HGL-induced HK-2 cell injury. **A,** Representative immunoblots of AKT, phospho-AKT (p-AKT), TGF-β1, CTSL, and NGAL in HK-2 cells cultured under control (Con) or high glucose and high lipid (HGL) conditions, with or without E64d. HSP90 was used as a loading control. **B-F,** Densitometric quantification of (B) total AKT, (C) p-AKT, **(D)** TGF-β1, **(E)** CTSL, and **(F)** NGAL. Protein levels were normalized to HSP90 and expressed relative to the control group. HGL increased TGF-β1, CTSL, and NGAL expression and reduced AKT phosphorylation; E64d partially reversed these changes. **G,** Representative Western blot images showing the expression of Tubulin, TGF-β1, p-AKT, AKT and NGAL in HK-2 cells exposed to normal control (Con), high glucose and high lipid (HGL), HGL plus KG (30 μM), HGL plus Iber (30 μM), or HGL plus Wig (10 μM) treatment. **H-L,** Quantitative densitometric analysis of Western blot results normalized to tubulin, demonstrating that KG significantly suppressed HGL-induced upregulation of CTSL and kidney injury related proteins. KG inhibited the downregulation of p-AKT, while the total AKT remained unchanged (n = 3). **M-O,** Relative mRNA expression levels of TGF-β1, NGAL, and AKT measured by RT-qPCR (n = 6). Data are shown as mean ± SEM from at least three independent experiments. Statistical analysis was performed using one-way ANOVA with Tukey’s post hoc test. *p < 0.05, **p < 0.01, ***p < 0.001.

Subsequently, we investigated the therapeutic potential of these compounds in a renal injury context by assessing their ability to alleviate renal injury in HK-2 cells exposed to HGL conditions. Exposure to HGL markedly activated injury pathways, resulting in substantial increases in CTSL, TGF-β1, and the kidney injury marker NGAL compared with the control group (*p* = 0.006, *p* = 0.012, and *p* = 0.017, respectively). In parallel, phosphorylated AKT (p-AKT) was markedly reduced (p < 0.001), whereas total AKT expression remained unchanged (Fig 6G–6L).

To verify that the HGL-induced injury model was responsive to CTSL inhibition, E64d was included as a positive control. Compared with the HGL group, E64d treatment significantly reduced the expression of CTSL (*p* = 0.043), NGAL (*p* = 0.042), and TGF-β1 (*p* = 0.001). In addition, E64d restored p-AKT levels (*p* = 0.012) without affecting total AKT expression. These findings confirmed that pharmacological inhibition of CTSL could partially reverse HGL-induced injury responses in HK-2 cells (Fig 6A–6F).

Having established that CTSL inhibition by the positive control E64d alleviated HGL-induced injury, we next evaluated whether the three AI-identified CTSL inhibitors could produce similar renoprotective effects under the same experimental conditions. Among the three compounds, KG exhibited the most pronounced protective effect against HGL-induced cellular injury. KG treatment substantially attenuated HGL-induced increases in CTSL, TGF-β1, and NGAL expression while partially restoring p-AKT levels (Fig 6G–L). These changes were statistically significant (CTSL, *p* = 0.009; TGF-β1, *p* = 0.02; NGAL, *p* < 0.001; p-AKT, *p* = 0.03). Consistent with the Western blot findings, RT-qPCR analysis showed that KG significantly reduced NGAL mRNA expression compared with the HGL group (*p* < 0.001; Fig 6M–6O). Notably, the overall magnitude of the protective response elicited by KG was comparable to that observed with the positive control E64d. Together, these findings indicate that KG exerts robust cytoprotective effects and may represent a promising lead compound for the treatment of renal injury.

Treatment with Iber effectively counteracted the HGL-induced renal injury. Western blot analysis revealed that Iber administration significantly suppressed the protein expression of CTSL (*p* = 0.023) and the renal injury marker NGAL (*p* < 0.001) (Fig 6G and 6J). RT-qPCR results demonstrated that Iber treatment markedly downregulated the mRNA transcripts of NGAL compared to the HGL-challenged group (*p* = 0.04) (Fig 6N). These findings confirm that Iber possesses renoprotective properties.

Treatment with Wig also exhibited protective effects against HGL-induced renal cellular injury, albeit with lower potency compared to KG and Iber. Specifically, Wig treatment significantly reduced CTSL expression (p = 0.034), and showed a downward trend for NGAL and TGF-β1 relative to the HGL-challenged group without reaching statistical significance for the latter two markers (Fig 6H and 6J). RT-qPCR results demonstrated that Wig treatment markedly downregulated the mRNA transcripts of NGAL compared to the HGL-challenged group (*p* = 0.03) (Fig 6N). While Wig demonstrated clear anti-renal injury potential and favorable binding affinity in MD simulations, its overall renoprotective efficacy was less pronounced than that of KG and Iber.

In alignment with the in vitro enzymatic assays, KG, Iber, and Wig all demonstrated protective efficacy against HGL-induced kidney injury responses in renal cells. Specifically, KG exhibited the most potent and comprehensive therapeutic effect, restoring p-AKT levels and suppressing renal injury factors (TGF-β1 and NGAL) to a greater extent than Iber and Wig. While Iber and Wig showed clear renoprotective properties, as evidenced by significant reductions in the mRNA levels of NGAL expression, their overall cellular efficacy was less pronounced than that of KG. These findings highlight KG as a particularly strong CTSL-targeted candidate for mitigating HGL-driven renal injury.

Notably, although KG, Iber, and Wig exhibited similar inhibitory potency against CTSL in the enzymatic assays, their protective effects in HK-2 cells differed. To investigate the potential basis for this discrepancy, we compared their cellular responses and intracellular accumulation profiles. KG markedly reduced CTSL-associated injury markers, while the positive control E64d produced similar protective effects, supporting the functional relevance of CTSL inhibition in this model.

LC-MS analysis further revealed differences in intracellular accumulation among the compounds (S6 Fig), suggesting that variation in cellular exposure may contribute to their differing activities, but does not fully explain them.

Together, these results suggest that while CTSL inhibition contributes to the observed protective effects, additional mechanisms or compound-specific properties are likely involved, particularly for KG.

### 3.5. Enzyme kinetics test

**Fig 7:**
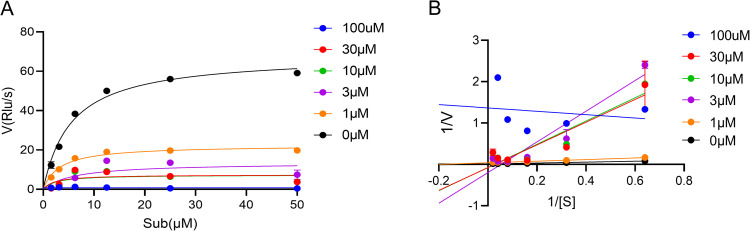
Enzyme kinetics results. **A-B,** Enzymatic kinetics of CTSL. The initial velocity (A) and Lineweaver-Burk plot (B) of the CTSL-catalyzed reaction were determined at various concentrations of the substrate in the presence of different concentrations of KG.

Enzyme kinetics provides a quantitative basis for evaluating the inhibitory potency and mechanistic characteristics of bioactive compounds. By determining key kinetic parameters such as the K_m_ and V_max_, different modes of inhibition—competitive, noncompetitive, uncompetitive, or mixed—can be distinguished. Such evaluation is critical for elucidating enzymatic control mechanisms and informing the structure-guided development of more selective and efficacious therapeutic agents. In the present study, KG was identified as the most potent CTSL inhibitor and was therefore subjected to detailed kinetic characterization. To further define its mode of action, we performed enzyme kinetic analyses to characterize the inhibition modality of KG. At lower concentrations (1–30 μM), KG reduced V_max_ without markedly affecting K_m_, consistent with a noncompetitive-like inhibition pattern. At higher concentration (100 μM), both K_m_ and V_max_ decreased, indicating an increased contribution of inhibitor binding to the enzyme–substrate complex. Rather than representing a discrete shift in inhibition mode, these results are more consistent with a mixed inhibition mechanism, in which KG interacts with both the free enzyme and the enzyme–substrate complex with different affinities (Fig 7A–7B). The calculated kinetic parameters are summarized in Table 2.

**Table 2:** Kinetic parameters of CTSL in the presence of KG.

Concentration(μM)	K_m_(μM)	V_max_ (Rlu/min)
0	5.6 ± 0.2	68.2 ± 0.3
6.25	3.3 ± 0.01	22.3 ± 0.04
12.5	6.3 ± 1.5	13.5 ± 2.0
25	2.5 ± 0.3	7.3 ± 0.4
50	2.3 ± 0.1	7.4 ± 0.4
100	0.1 ± 0.01	0.8 ± 0.01

## 4. Discussion

In this study, we developed an AI-guided natural product discovery approach that successfully identified KG as a novel and potent inhibitor of CTSL, demonstrating strong therapeutic potential for kidney injury.

Aberrant activation of CTSL in the diabetic kidney is a well-documented pathogenic event that contributes to podocyte injury and foot-process effacement via proteolytic cleavage of cytoskeletal regulators such as dynamin, thereby compromising the glomerular filtration barrier and promoting proteinuria [5]. In experimental models of diabetic nephropathy, CTSL activity has also been causally linked to early albuminuria and to downstream activation of proteases (e.g., heparanase) that promote ECM remodeling and glomerular injury [6].

Our data extend these pathophysiological insights by showing that pharmacological CTSL inhibition can directly counteract processes central to renal injury. KG displayed exceptional inhibitory potency, consistent with a mixed inhibition mechanism by occupying the catalytic pocket and preventing substrate access. Moreover, the stable KG-CTSL complex observed in our 100-ns molecular dynamics simulations, together with its favorable binding free energy, provides a structural basis for the observed potent inhibitory activity and selectivity [37].

Beyond its enzymatic inhibition, KG exhibited pronounced renoprotective effects in a cellular model mimicking diabetic glucolipotoxic stress. In human renal cells, HGL conditions markedly induced CTSL expression and injury-related transcriptional programs. Conversely, KG treatment significantly abrogated these effects, downregulating key markers such as TGF-β1 at both mRNA and protein levels, thereby mitigating pathological ECM deposition—a hallmark of renal injury progression [38]. We also found that KG restored AKT phosphorylation reduced by HGL in the cellular model. It plays a pivotal role in renal pathogenesis and is strongly associated with cellular dysfunction [39].

Moreover, previous studies have shown that cathepsin-dependent proteolysis can intersect with kinase signaling pathways in podocytes and other renal cell types, providing a mechanistic rationale by which CTSL inhibition may restore downstream signaling homeostasis [40]. Consistent with these mechanistic insights, KG administration significantly downregulated TGF-β1, a key mediator of renal injury, alongside a concurrent reduction in the cellular stress marker NGAL. These findings underscore the therapeutic potential of KG in ameliorating renal impairment and slowing the trajectory of the disease.

Recent evidence highlights the critical role of immune cells, particularly macrophages, in the progression of liver and renal injury. Single-cell RNA sequencing studies have demonstrated that macrophage metabolic reprogramming is a key driver of non-alcoholic fatty liver disease (NAFLD) [41]. Notably, CTSL is not only expressed in epithelial cells but is also functionally active in immune cell populations, suggesting that its inhibition may exert broader effects beyond epithelial compartments. Future studies incorporating co-culture models, in vivo validation, or single-cell transcriptomic approaches will be necessary to determine whether macrophage-mediated mechanisms contribute to the renoprotective effects of KG.

A notable strength of this study lies in its AI-guided screening strategy. The graph-based deep learning model (ROC-AUC = 0.93) demonstrated excellent enrichment efficiency from a large phytochemical library, substantially reducing the experimental workload compared with conventional high-throughput screening approaches. Recent studies have shown that well-optimized machine learning and deep learning workflows can markedly enhance virtual screening accuracy and hit discovery rates, particularly when integrated with complementary filtering strategies [42]. To minimize artifacts commonly encountered in natural product screening—such as fluorescence interference and frequent hitters—we combined AI prediction with MD and a rigorous fluorescence-interference counter-screen, an approach consistent with current best practices for minimizing false positives in natural product-based discovery [43].

Although KG, Iber, and Wig all inhibited CTSL activity in biochemical assays and displayed stable binding conformations in MD simulations, all three compounds significantly ameliorated HGL-induced pathological alterations in renal cells. Notably, however, KG produced the most pronounced protective effects, demonstrating a greater attenuation of injury-associated markers compared with Iber and Wig. This divergence in the magnitude of cellular efficacy, despite broadly comparable enzymatic inhibition profiles, prompted us to further examine the underlying determinants of compound activity in the cellular context.

Although CTSL inhibition is clearly functionally relevant—as supported by the observation that the reference inhibitor E64d recapitulated key protective phenotypes—our additional analyses suggest that CTSL inhibition alone does not fully account for the differential cellular effects observed. In particular, intracellular exposure profiling revealed differences in compound accumulation, indicating that variability in cellular uptake or retention may partially contribute to efficacy differences. Nevertheless, these factors are unlikely to completely explain the observed divergence, suggesting that KG may exert additional CTSL-independent effects or engage downstream signaling pathways more effectively than Iber and Wig. First, flavonoid glycosides exhibit markedly different cellular uptake and hydrolysis characteristics; some glycosides have minimal intracellular accumulation and lack functional effects despite favorable in vitro enzyme inhibition [44]. Second, the intracellular exposure of kaempferol derivatives is modulated by phase II metabolic enzymes and efflux transporters, which influence their pharmacodynamic effectiveness in cellular contexts [45]. Third, the reported cellular actions of Iber and Wig are largely limited to antiproliferative and pro-apoptotic effects in cancer cell lines, rather than modulation of the injury-related and inflammatory pathways relevant to kidney diseases, suggesting divergent cellular targets [46].

The concentration-dependent changes in the apparent inhibition pattern of KG are consistent with a mixed inhibition mechanism. Such behavior can arise when an inhibitor interacts with both the free enzyme and the enzyme–substrate complex with different affinities, leading to noncompetitive-like features at lower concentrations and increased contributions from enzyme–substrate complex binding at higher concentrations. This pattern may also reflect the conformational flexibility of CTSL, whereby ligand binding stabilizes different enzyme states across the concentration range.

Although all three compounds exhibited enzyme inhibitory activity and favorable in silico binding profiles, KG’s broader bioactivity spectrum may contribute to its superior cellular efficacy. In contrast, Iber and Wig lack robust literature supporting such pleiotropic cellular effects, which may explain why they failed to ameliorate pathological markers in cell models despite inhibiting CTSL [47].

KG consistently exhibited potent inhibitory activity and effectively attenuated injury-associated cellular responses, while showing no detectable cytotoxicity in renal cells at therapeutically relevant concentrations—an essential feature for agents intended for chronic administration in kidney injury. Such a pharmacological profile is particularly advantageous in this context, where the therapeutic objective is the sustained modulation of maladaptive cellular stress and inflammatory processes rather than acute cytotoxic elimination of affected cells. Compounds suitable for long-term use in patients with renal impairment must demonstrate minimal off-target toxicity, stable pharmacodynamic properties, and durable regulation of injury-related signaling pathways. The absence of cytotoxicity observed in our renal cell-based assays suggests that KG may have favorable safety properties and warrants further investigation in kidney injury.

In a broader context, KG, a bioactive compound derived from natural sources, fits within the emerging framework of “food–medicine homology,” which emphasizes the dual nutritional and pharmacological roles of dietary compounds in modulating metabolic disorders. Recent studies have highlighted that such compounds can influence key pathogenic processes, including metabolic dysregulation and inflammation, thereby contributing to the prevention or attenuation of diabetic complications [48–50]. Although the present study focuses on cellular mechanisms rather than dietary or in vivo effects, our findings provide mechanistic support for the potential integration of KG-related natural products into future nutraceutical or dietary intervention strategies targeting kidney injury.

Taken together, the combination of potent CTSL inhibition, absence of detectable cytotoxicity, and its natural product origin supports KG as a CTSL-targeting compound with potential relevance for kidney injury. Further studies are warranted to more fully characterize its biological activity and to clarify its potential applicability in this context.

## 5. Conclusions

In summary, this study establishes a deep learning-assisted discovery framework for identifying CTSL inhibitors and provides mechanistic insight into ligand-enzyme interactions. By integrating enzymatic assays and molecular dynamics simulations, we identified KG, Iber, and Wig as potent CTSL inhibitors with stable binding within the catalytic pocket. Moreover, *in vitro* experiments showed that KG attenuated high glucose and high lipid induced inflammatory and injury responses in human renal cells, supporting its potential as a lead compound for kidney diseases. Collectively, these findings underscore the value of AI-assisted strategies in accelerating drug discovery.

## Supporting information

S1 Table Training dataset information, including the compound information and activity labels used for training, comprising 2062 compounds annotated as active CTSL inhibitors and 58,005 compounds annotated as inactive. (CSV)

S2 Table Integrated ranking and experimental validation results of the top 200 candidate natural compounds identified through AI-based prediction and molecular docking. The table summarizes the catalog number, compound name, CTSL activity, Chemprop-predicted activity, molecular docking score, Chemprop rank, docking rank, total score, and total ranking for the top 200 screened compounds. CTSL activity represents the percentage inhibition of CTSL enzymatic activity at a concentration of 100 μM.
(XLSX)

S1 Fig Effects of E64d on HK-2 cell viability and CTSL enzymatic activity. **A,** Cell viability of HK-2 cells following treatment with E64d at the indicated concentrations, measured by CCK-8 assay. **B,** Inhibition of CTSL enzymatic activity by E64d in the fluorogenic assay.
(TIF)

S2 Fig The enzyme activity curve corresponding to the half maximal inhibitory concentration (IC_50_) determination. **A-I,** Nine natural CTSL inhibitors—Mulberrin (A), Iber (B), (-)-Alkannin (C), 8-Methylsulfinyloctyl isothiocyanate (D), KG (E), γ-Mangostin (F), Wig (G), Glycyrrhisoflavone (H), and Glabrol (I)—that showed > 90% inhibition of CTSL activity at 100 μM in the initial screen were further tested to determine their half-maximal inhibitory concentrations (IC_50_) in a cell-free enzymatic system.
(TIF)

S3 Fig Equilibrium results from molecular dynamics simulations. **A-C,** Dynamic variation of potential energy (A), pressure (B) and temperature (C) observed in the MD simulation of CTSL with KG. **D-F,** Dynamic variation of potential energy (D), pressure (E) and temperature (F) observed in the MD simulation of CTSL with Iber. **G-I,** Dynamic variation of potential energy (G), pressure (H) and temperature (I) observed in the MD simulation of CTSL with Wig. **J,** ΔG_bind_ values were calculated at 1 ns intervals over the 100 ns MD trajectories. Red represents KG, black represents Iber, and blue represents Wig. The profiles show the temporal variation of MM/PBSA binding free energy during the simulations.
(TIF)

S4 Fig The CCK8 assay of three natural compounds and E64d across a concentration range of 1–100 μM. **A,** KG; **B,** Iberverin; **C,** Wighteone; **D,** E64d.
(TIF)

S5 Fig Enzyme inhibitory kinetics of KG at different concentrations. **A-F,** The kinetic curves of enzyme activity corresponding to the KG concentrations of 100 μM (A), 30 μM (B), 10 μM (C), 3 μM (D), 1 μM (E), and 0 μM (F).
(TIF)

S6 Fig Intracellular accumulation of compounds in HK-2 cells assessed by LC–MS. **A,** Intracellular accumulation of compounds in HK-2 cells measured by LC–MS, performed to evaluate compound exposure levels and address potential differences in cellular uptake.
(TIF)

S1 Materials The following supplementary files are provided to support reproducibility of the molecular docking and GROMACS molecular dynamics simulation workflow. (ZIP)

S1 Raw Image Uncropped original western blot images corresponding to Fig 6A and Fig 6G. The red boxes indicate the regions used in the main figures of the manuscript. Images were acquired by chemiluminescence detection. AKT and phospho-AKT were analyzed using parallel gels loaded with identical protein samples.
(PDF)
