# Supplementary material for: AI-guided identification of natural CTSL inhibitors with therapeutic potential for renal injury
Source: PLoS Comput Biol. 2026 Jul 10;22(7):e1014464. doi: 10.1371/journal.pcbi.1014464 (PMC13399510; doi:10.1371/journal.pcbi.1014464)
Supplement: S1 Materials — (ZIP) [file pcbi.1014464.s009.zip › Supplementary Materials/Legends.docx]

| **File** | **Legend** |
| --- | --- |
| ctsl.pdb | Prepared CTSL receptor structure used for molecular docking and molecular dynamics simulations. |
| em.mdp | GROMACS MDP parameter file used for energy minimization. |
| iber.mol2 | MOL2 structure file of Iber used as ligand input for docking and molecular dynamics simulation. |
| iber_topol.top | GROMACS topology file for the Iber–CTSL complex used in molecular dynamics simulations. |
| ions.mdp | GROMACS MDP parameter file used to generate the ion-addition input for system neutralization. |
| kg.mol2 | MOL2 structure file of KG used as ligand input for docking and molecular dynamics simulation. |
| kg_topol.top | GROMACS topology file for the KG–CTSL complex used in molecular dynamics simulations. |
| md.mdp | GROMACS MDP parameter file used for the production molecular dynamics simulation. |
| npt.mdp | GROMACS MDP parameter file used for NPT equilibration. |
| nvt.mdp | GROMACS MDP parameter file used for NVT equilibration. |
| wig.mol2 | MOL2 structure file of Wig used as ligand input for docking and molecular dynamics simulation. |
| wig_topol.top | GROMACS topology file for the Wig–CTSL complex used in molecular dynamics simulations. |
