# Supplementary material for: AI-guided identification of natural CTSL inhibitors with therapeutic potential for renal injury
Source: PLoS Comput Biol. 2026 Jul 10;22(7):e1014464. doi: 10.1371/journal.pcbi.1014464 (PMC13399510; doi:10.1371/journal.pcbi.1014464)
Supplement: S1 Raw Image — The red boxes indicate the regions used in the main figures of the manuscript. Images were acquired by chemiluminescence detection. AKT and phospho-AKT were analyzed using parallel gels loaded with identical protein samples. (PDF) [file pcbi.1014464.s010.pdf]

Figure 6A – Original Uncropped Western Blot Images

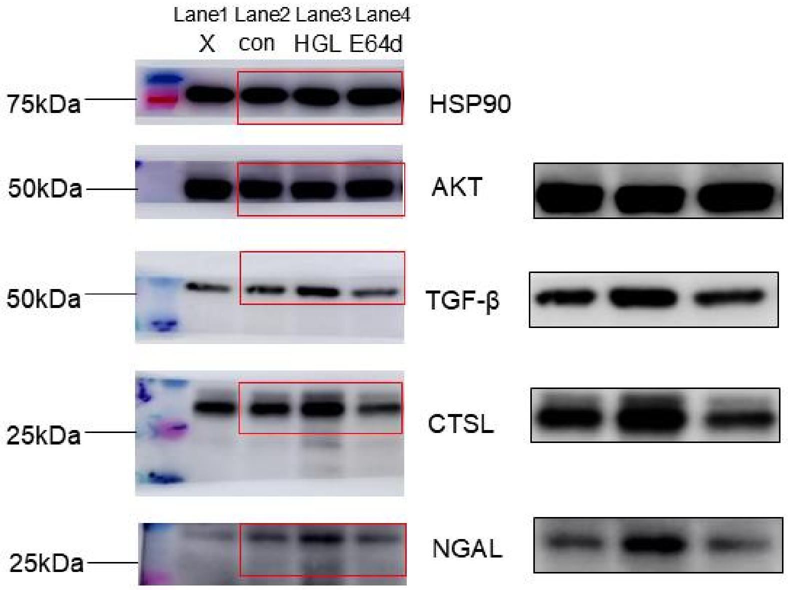

Cell model:
HK-2 cells cultured under control (Con) or high glucose and high lipid (HGL) conditions, with or without E64d treatment.

Image acquisition:
Chemiluminescence detection.

The region enclosed by the red box corresponds to the cropped image presented in Figure 6A of the manuscript.

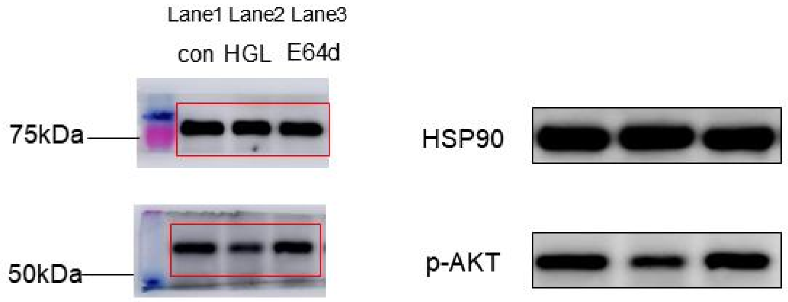

Note:
AKT and phospho-AKT were analyzed using parallel gels loaded with identical protein samples.

Figure 6G – Original Uncropped Western Blot Images

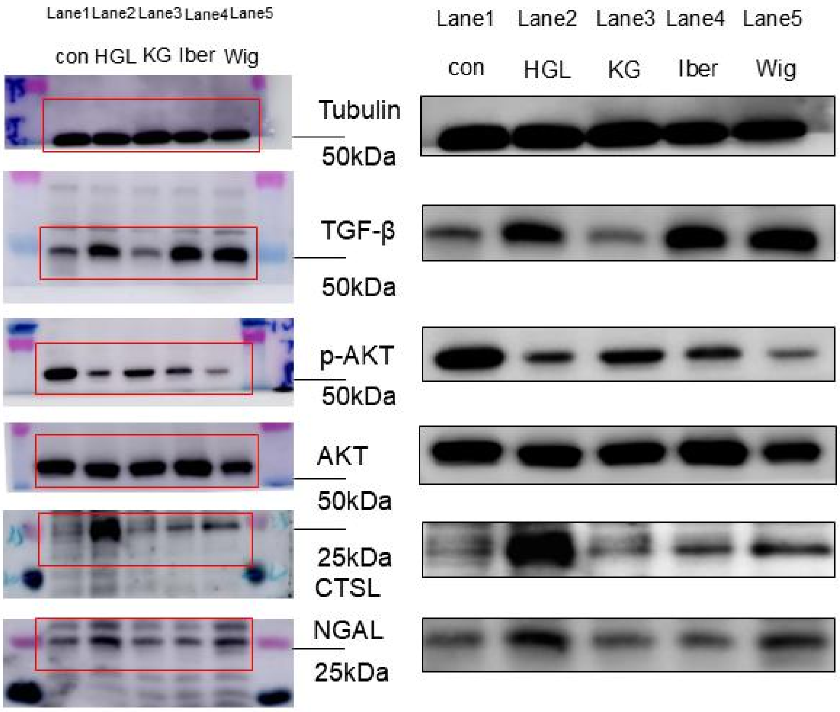

Cell model:
HK-2 cells exposed to normal control (Con), high glucose and high lipid (HGL), HGL + KG (30 μM), HGL + Iber (30 μM), or HGL + Wig (10 μM).

Image acquisition:
Chemiluminescence detection.

The region enclosed by the red box corresponds to the cropped image presented in Figure 6G of the manuscript.

Note:
AKT and phospho-AKT were analyzed using parallel gels loaded with identical protein samples.
